# Seasonal dietary shifts alter the gut microbiota of a frugivorous lizard *Teratoscincus roborowskii* (Squamata, Sphaerodactylidae)

**DOI:** 10.1002/ece3.10363

**Published:** 2023-08-02

**Authors:** Wei‐Zhen Gao, Yi Yang, Lei Shi

**Affiliations:** ^1^ College of Life Sciences Xinjiang Agricultural University Urumqi China

**Keywords:** 16S rRNA gene, ecological adaptation, gut microbiota, LC–MS metabolomics, *s*easonal dietary changes, *Teratoscincus roborowskii*

## Abstract

Seasonal dietary shifts in animals are important strategies for ecological adaptation. An increasing number of studies have shown that seasonal dietary shifts can influence or even determine the composition of gut microbiota. The Turpan wonder gecko, *Teratoscincus roborowskii*, lives in extreme desert environments and has a flexible dietary shift to fruit‐eating in warm seasons. However, the effect of such shifts on the gut microbiota is poorly understood. In this study, 16S rRNA sequencing and LC–MS metabolomics were used to examine changes in the gut microbiota composition and metabolic patterns of *T. roborowskii*. The results demonstrated that the gut microbes of *T. roborowskii* underwent significant seasonal changes, and the abundance of phylum level in autumn was significantly higher than spring, but meanwhile, the diversity was lower. At the family level, the abundance and diversity of the gut microbiota were both higher in autumn. Firmicutes, Bacteroidetes, and Proteobacteria were the dominant gut microbes of *T. roborowskii*. Verrucomicrobia and Proteobacteria exhibited dynamic ebb and flow patterns between spring and autumn. Metabolomic profiling also revealed differences mainly related to the formation of secondary bile acids. The pantothenate and CoA biosynthesis, and lysine degradation pathways identified by KEGG enrichment symbolize the exuberant metabolic capacity of *T. roborowskii*. Furthermore, strong correlations were detected between metabolite types and bacteria, and this correlation may be an important adaptation of *T. roborowskii* to cope with dietary shifts and improve energy acquisition. Our study provides a theoretical basis for exploring the adaptive evolution of the special frugivorous behavior of *T. roborowskii*, which is an important progress in the study of gut microbes in desert lizards.

## Graphical abstract

Seasonal dietary shifts led to the alterations of the gut microbiota in *T. roborowskii*. On the one hand, the core flora such as Firmicutes and Bacteroidetes have stable relative abundance, and on the other hand, other flora such as Proteobacteria and Lachnospiriaceae have flexible seasonal variation patterns. The correlation studies with metabolomics also indicate the stable state and dynamic changes of gut microbiota, which may be to adapt to the changes of food and meet the functional requirements of food digestion and metabolism. Our study provides a theoretical basis for exploring the adaptive evolution of *T. roborowskii* to special frugivorous behaviors.

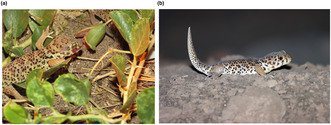

## 1 INTRODUCTION

The intestinal structure of animals satisfies the requirements for food digestion and nutrient absorption. It also maintains the homeostasis of gut microbes (Sommer & Bäckhed, 2013). Complex interactions occur in the gut microbial ecosystem between the host and gut microbes (McFall‐Ngai et al., 2013). The gut microbiota plays an important role in the intestinal immune system, energy metabolism (Sommer & Bäckhed, 2013), reproductive activity (MacLeod et al., 2022), and longevity (Kim & Benayoun, 2020) of the host.

Numerous factors, such as dietary structure, host physiological status, and captivity (Jiang et al., 2017; Montoya‐Ciriaco et al., 2020; Trevelline et al., 2019; Zhou et al., 2020) can lead to changes in gut microbial composition, structure, and function (Suzuki & Worobey, 2014). Seasonal environmental fluctuations and their effects on host physiology and ecology can significantly alter the gut microbiota of animals. For example, the gut microbiota of Siberian flying squirrels (*Pteromys volan sorii*) varies seasonally on an ecological scale (Liu et al., 2019). In addition, seasonal physiological changes associated with hibernation alter gut microbiota in greater horseshoe bats (*Rhinolophus ferrumequinum*) and brown bears (*Ursus arctos*; Sommer et al., 2016; Xiao et al., 2019). Studies on wood mice (*Apodemus sylvaticus*), plateau pikas (*Ochotona curzoniae*), brown frogs (*Fejervarya limnocharis*), black howler monkeys (*Alouatta pigra*), and giant pandas (*Ailuropoda melanoleuca*) have shown that the changes in animals microbiota composition that response to seasonal was driven by dietary shift (Amato et al., 2015; Fan et al., 2022; Huang & Liao, 2021; Maurice et al., 2015; Wu et al., 2017). Trophic niche expansion is an active adjustment of wildlife to seasonal and environmental changes (Guisan et al., 2014), and dietary shifts can affect or even determine the composition of animal gut microbes, which change dynamically with the season (Maurice et al., 2015).

At present, fecal metabolomics is widely considered a key tool for studying the relationship between hosts and their gut microbiota and has attracted extensive attention (Zierer et al., 2018). Metabolomics enables researchers to identify a large proportion of the metabolites present in a sample. These metabolites include host, microbial, and exogenous small molecules (Chung et al., 2018). Metabolomic analysis can reveal valuable information about the metabolic or physiological state of an organism at the time of sampling (Tran et al., 2020). For example, in wildlife research, the joint analysis of metabolomics and gut microbiota has revealed differences in the abundance and metabolic phenotypes of gut microbes in North China leopards (*Panthera pardus japonensis*) in captive and wild environments (Hua et al., 2020). The co‐metabolic patterns of the gut and microbes in mountain gorillas (*Gorilla beringei beringei*) and western lowland gorillas (*Gorilla gorilla gorilla*) indicate that dietary restriction is a potential factor driving specific changes in gut microbes. For example, increased consumption and fermentation of plant polysaccharides are associated with an increased abundance of Bacteroidetes and decreased abundance of Firmicutes. Lachnospiraceae and Erysipelotrichaceae were significantly associated with a high proportion of butyrate in feces. Carbohydrates in the diet are composed of soluble butyrogenic substrates (Gomez et al., 2016). The combined analysis of gut microbes and metabolomics has also become an effective approach for identifying relevant biomarkers that reveal complex changes in metabolic and biochemical pathways (Tran et al., 2020).

Significant differences in the composition, structure, and abundance of gut microbiota in different animal groups, such as mammals (Zhao et al., 2018), birds (Grond et al., 2018), and fish (Egerton et al., 2018), have been extensively studied. In recent years, the study of gut microbes in reptiles, including lizards has attracted considerable attention. Relevant studies have focused on changes in the patterns of gut microbes in slender anole lizards (*Anolis apletophallus*), western fence lizards (*Sceloporus occidentalis*), mongolian racerunners (*Eremias sargus*), Puerto Rican *Anolis* lizards, and mesquite lizards (*Sceloporus grammicus*) under the influence of climate anomalies, temperature, ecomorphs, altitude, and other factors (Moeller et al., 2020; Montoya‐Ciriaco et al., 2020; Ren et al., 2016; Williams et al., 2022; Zhang et al., 2022). In addition, in a study on crocodile lizards (*Shinisaurus crocodilurus*), northern grass lizards (*Takydromus septentrionalis*), and Puerto Rican *Anolis* lizards in captivity, captivity was found to significantly alter the gut microbiota of the lizards, and diet was considered the main factor responsible for this difference, which suggests that there is an extremely significant relationship between diet and gut microbiota in lizards (Ren et al., 2016; Tang et al., 2020; Zhou et al., 2020).

**FIGURE 1:**
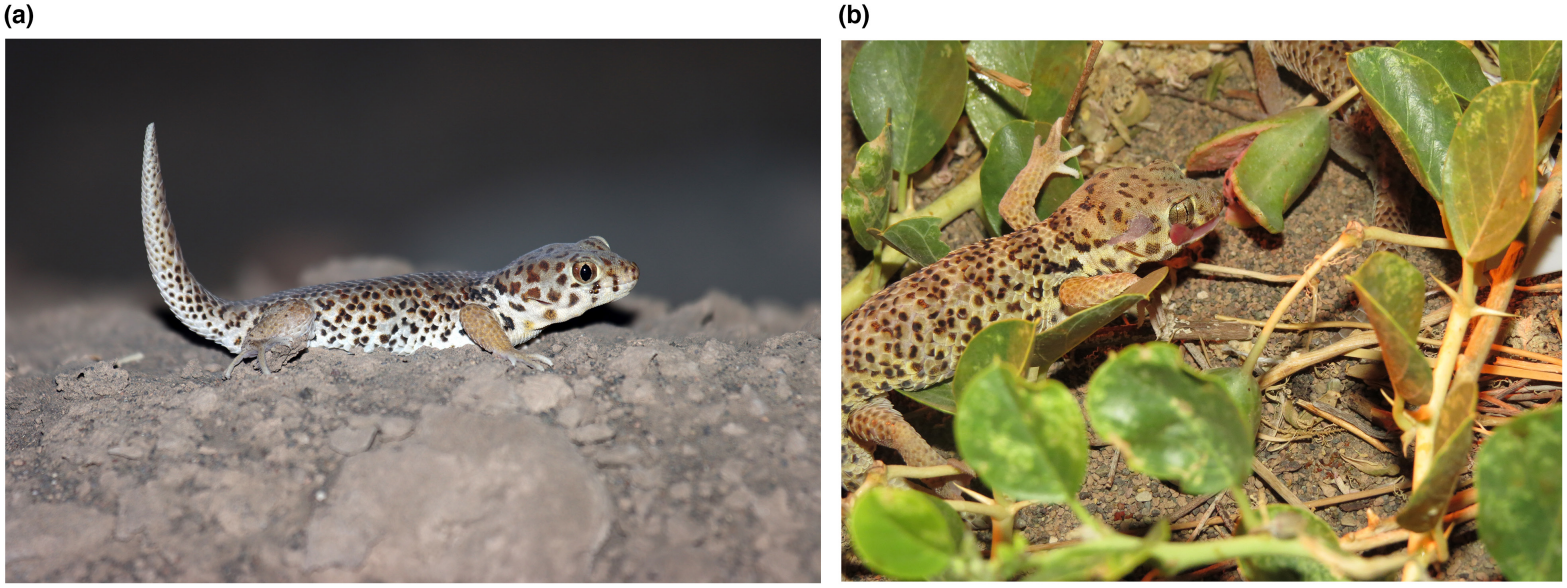
Turpan wonder gecko (*Teratoscincus roborowskii*) in the wild (a), *T. roborowskii* was feeding on caper fruit (*Capparis spinosa* L.) (b).

The Turpan wonder gecko (*Teratoscincus roborowskii*; Figure 1) is a walking gecko species endemic to the Turpan Depression in the Xinjiang Uyghur Autonomous Region of China (Shi et al., 2002, 2007). They live in extreme desert environments (Song et al., 2009; Zhou et al., 2019). In this area, the average elevation is only −95 ~ −76 m, the average annual precipitation is 16.4 mm, and the annual evaporation is 3000 mm. The extreme high temperature is 49.6°C, and the maximum surface temperature in summer can reach 80°C. Research on Turpan wonder geckos has mainly focused on foraging modes, activity rhythms, and seed dispersal (Song et al., 2017; Werner et al., 1997; Yang et al., 2021). Field observations and dietary analyses have shown that *T. roborowskii* feeds mainly on insects in spring, whereas the proportion of caper fruits consumed can reach 85% in summer and autumn, showing a significant seasonal shift in dietary habits (Liu et al., 2010; Yang et al., 2021). This seasonal dietary shift may be an important factor in *T. roborowskii* adaptation to harsh arid desert habitats.

The Turpan wonder gecko undergoes a long hibernation process annually; it enters hibernation in October and ends in late March of the following year. Hibernation lasts for approximately 6 months (Li et al., 2010). Fruit‐eating strategies are more likely to be deployed with seasonal variations in food availability to maximize prehibernation food intake and satisfy nutritional requirements (Naya et al., 2010; Robbins et al., 2012). Studies have shown that switching from insects to fruits is a common mechanism by which migrating birds accumulate fat for energy (Marshall et al., 2016). Gerbils (*Psammomys obesus*), which also live in desert environments, can convert a small amount of carbohydrates into endogenous fructose owing to the stimulation of a high‐salt diet, thereby aggravating the tendency toward obesity and effectively adapting to the lack of food (Kaiser et al., 2012). It is possible that Turpan wonder geckos rely on a dietary switch from insects to fruits to accumulate necessary fat reserves for hibernation. However, whether or not this dietary shift corresponds to a shift in microbiota and whether or not microbially‐mediated metabolism might be important for this dietary transition remains unclear. Here, we used 16S rRNA sequencing paired with LC–MS metabolomics to explore the changes in the gut microbiota and metabolic patterns of Turpan wonder geckos during spring and autumn.

## 2 MATERIALS AND METHODS

### 2.1 Animal and feces collection

*Teratoscincus roborowskii* individuals were captured from a desert in the suburbs of Turpan between May 2021 and September 2021. The capture site was located near the Turpan Eremophyte Botanic Garden, located in the Turpan Basin of the Xinjiang Uyghur Autonomous Region of China (40°51′ N, 89°11′ E). We shined a flashlight on the ground and determined the location of *T. roborowskii* by the bright spots generated through the reflection of the light from its eyes, at the peak time of *T. roborowskii* activity (23:00–1:00). The experiment was divided into two groups: (1) 11 lizards were captured in spring (spring group; SG) and 23 complete fresh fecal pellets were collected during the fasting period; (2) 15 lizards were captured in autumn (autumn group; AG), and 21 complete fresh fecal pellets were collected during the fasting period (Table S1).

After capture, the Turpan wonder geckoes were recorded for sex and other basic information. The spring group's snout‐vent lengths (SVLs) ranged from 70.36 to 94.72 mm, with a mean of 82.27 ± 2.91 mm; body masses ranged from 12.44 to 24.71 g, with a mean of 17.72 ± 1.38 g. The autumn group's SVLs ranged from 69.36 to 88.09 mm, with a mean of 78.77 ± 1.46 mm; body masses ranged from 11.0 to 20.00 g, with a mean of 15.04 ± 0.68 g. Subsequently, the lizards were individually maintained in 30 × 21 × 15.5 cm (L × W × H) plastic rearing boxes, cleaned using 75% ethanol. A non‐invasive fecal sampling method was used to check for excretion every 2 h to ensure the timely collection of fresh fecal samples. Complete fecal samples were collected in sterile cryovials using sterilized tweezers. Samples were immediately marked and snap‐frozen in liquid nitrogen, then transferred to a −80°C freezer for subsequent experimental operations. Our previous study showed that the transit time of food through the digestive tract of *T. roborowskii* was up to 15 days (Yang et al., 2021), therefore, fecal collection was performed while no food was provided for 15 days. After the experiment, all the animals were in good physiological condition and were treated in strict accordance with the relevant regulations on animal welfare. All experiments and animal handling were conducted according to research protocols approved by the Animal Welfare and Ethics Committee of Xinjiang Agricultural University.

### 2.2 DNA extraction and PCR amplification

In the spring group, feces were randomly selected from 2 to 3 individuals and mixed immediately to represent one sample, whereas in the autumn group, feces were randomly selected from 3 to 4 individuals. Four mixed samples were obtained from each group (Zhou et al., 2020). Total bacterial DNA was extracted from fecal samples using a TGuide S96 Magnetic Soil and Stool DNA Kit (TIANGEN; Cat. No. DP812). The quantity and quality of the extracted DNA were measured using a microplate reader (Biotek Synergy HTX; Agilent Technologies, Inc.) and 1.8% agarose gel electrophoresis, respectively.

Using the total DNA that passed the test as a template, the V3–V4 hypervariable regions of the microbial 16S rRNA gene were amplified by polymerase chain reaction (PCR) using the primers 27F (5′‐AGRGTTTGATYNTGGCTCAG‐3′) and 1492R (5′‐TASGGHTACCTTGTTASGACTT‐3′; Kim et al., 2011). PCR was performed in the reaction system with a total volume of 30 μL, supplemented with 3 μL DNA primer, 15 μL of KOD OneTM PCR Master Mix, 10.5 μL NFW, and 1.5 μL genomic DNA. The PCR conditions were as follows: 95°C for 2 min; 25 cycles at 98°C for 10 s, 55°C for 30 s, and 72°C for 90 s; and extension at 72°C for 2 min. The SMRTbell Template Prep Kit (PacBio) was used for damage repair, terminal repair, and joining of the mixed products, and AMpure PB (PacBio) beads were used for purification and recovery to obtain a sequencing library (SMRT Bell). Qualified products were sequenced using PacBio Sequel II at Biomarker Technologies Co., Ltd.

Raw subreads were calibrated to obtain circular consensus sequencing (CCS; SMRT Link, version 8.0), and Lima (v1.7.0) software was used to identify the CCS sequences of different samples through barcode sequences and remove chimeras to obtain Effective‐CCS sequences. Operational taxonomic units (OTUs) were clustered with a 97% similarity cutoff using UPARSE (version 10.0; Edgar, 2013). Detailed sequencing data of each sample are shown in Table S2.

Alpha diversity index analysis was performed using QIIME2 (version 2020.6) software (Bolyen et al., 2019), and the Wilcoxon rank‐sum test was used to compare community diversity indices (Ace richness estimator and Shannon–Wiener index; Prehn‐Kristensen et al., 2018; Schloss et al., 2011).

The dimensionality reduction of the data was based on the Bray‐Curtis distance matrix using principal coordinate analysis (PCoA) and nonmetric multidimensional scaling (NMDS). This was used to observe differences in the gut microbial community structure (Oksanen et al., 2013). In addition, Permutational multivariate analysis of variance (PERMANOVA; McArdle & Anderson, 2001) and analysis of similarities (ANOSIM; Anderson, 2001) were conducted using the R v3.1.1 package “vegan” (v2.3‐0; Oksanen et al., 2015) to assess the significance of the difference of the microbiota structures among groups.

Using SILVA 132 (Quast et al., 2012) as the reference database, the community species composition analysis of the two groups of experiments was performed at various levels: phylum, order, family, genus, and species. Linear discriminant effect size (LEfSe) analysis was used to screen microorganisms with large differences as potential markers (Segata et al., 2011), the significance of different species was determined using Matestats software, and differential strains were screened according to the criteria of LDA > 4, q < 0.05. PICRUSt2 (Douglas et al., 2019) software was used to compare the 16S sequencing data to obtain species composition information based on the Kyoto Encyclopedia of Genes and Genomes (KEGG) database (Kanehisa & Goto, 2000); the gene functions corresponding to the sequencing data were obtained and corresponded to the KEGG pathways. The significance of the functional pathways in the two groups of samples was compared using the *t*‐test in STAMP (Parks et al., 2014), and the *p*‐value threshold of .05.

### 2.3 LC–MS metabolomics detection

The feces used for LC–MS are the same as the samples used for DNA extraction. A fecal sample (50 mg) was weighed, and 1000 μL of the extract containing an internal standard (1000:2) (methanol acetonitrile: water = 2:2:1, internal standard concentration 2 mg/L) was added, and the sample vortexed for 30 s. Subsequently, magnetic beads were added, ground at 45 Hz for 10 min, sonicated in an ice‐water bath for 10 min, then let stand for 1 h, and centrifuged at 4°C, 13,400 *g* for 15 min. A 500 mL aliquot was placed in a centrifuge tube and dried under vacuum. After drying, 160 μL of the extract (acetonitrile:water = 1:1) was added, vortexed for 30 s, sonicated for 10 min in an ice water bath, and centrifuged at 13,400 *g* for 15 min at 4°C. Finally, 120 μL of the supernatant was collected, and 10 μL taken from each sample to be mixed into QC samples for machine testing.

Metabolites in fecal samples were detected in positive and negative ion modes and separated using a Waters Acquity 1‐Class PLUS ultraperformance liquid chromatography system (XevoG2‐XS QTOF high‐resolution mass spectrometer; Waters Corp.). Primary and secondary mass spectrometry data were collected in MSe mode under the control of an acquisition software (MassLynx V4.2; Waters). In each data acquisition cycle, dual‐channel data acquisition was performed simultaneously at both low and high collision energies. The low collision energy was 2 V, the high collision energy range was 10–40 V, and the scI ion source was as follows: capillary voltage, 2000 V (positive ion mode) or –1500 V (negative ion mode); cone voltage, 30 V; ion source temperature, 150°C; desolvent gas temperature, 50°C; backflush gas flow rate, 50 L/h; desolventizing gas flow rate, 800 L/h (Wang et al., 2020).

The raw data collected using MassLynx V4.2 (Waters Corp.) were processed using Progenesis QI software for peak extraction, peak alignment, and other data processing operations, based on the Progenesis QI (Waters Corp.) software online METLIN database (Smith et al., 2005) and Biomark's self‐built library for identification; at the same time, theoretical fragment identification and mass deviation were within 100 ppm.

### 2.4 Quality control and data analysis of fecal metabolite detection

Principal component analysis (PCA) was performed on the metabolite data and the OPLS‐DA model was calculated using the R (3.3.2) (R Core Team, 2013) package “ropls” to visualize sample clustering (Thevenot, 2016). Differential metabolites were screened by combining the fold difference, *p*‐value of the *t*‐test, and the VIP value of the OPLS‐DA model. The screening criteria were FC > 1, *p*‐value < .05, and VIP > 1. The cluster profiler used the hypergeometric test method to perform enrichment analysis on the annotation results of the differential KEGG metabolites and screened representative metabolic pathways for impact value and significance as the enrichment criteria.

Metabolome data were preprocessed by UV scaling using R (v 3.6.1), and metabolite data were dimensionalized using hierarchical cluster analysis (HCA). The metabolites were divided into different metabolite clusters, and data with a correlation *p*‐value satisfying CCP < 0.05 were retained. The NbClust package selected the best number of metabolite clusters, the R hclust function performed clustering, and the metabolite clusters were determined using the R cutree function. The Hmisc package performed correlation analysis, and the pheatmap package performed correlation map visualization.

## 3 RESULTS

### 3.1 Composition of gut microbiota

**FIGURE 2:**
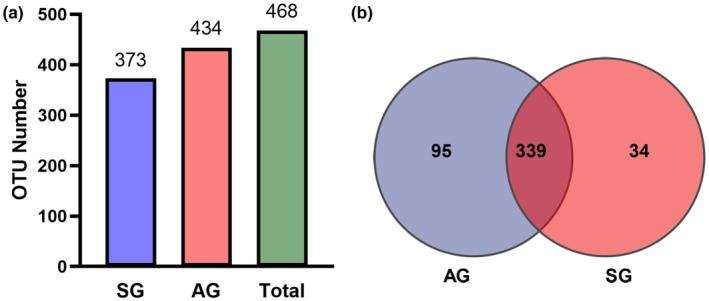
Bar diagram (a) and Venn plot (b) of gut microbiota OTU between SG (spring) and AG (autumn) groups from *Teratoscincus roborowskii*.

In total, 468 OTUs were divided into spring (SG) and autumn (AG) groups (Figure 2a). Among these, there were 373 OTUs in SG, 434 in AG, and 339 in both groups (Figure 2b).

### 3.2 Analysis of alpha diversity of gut microbiota

**FIGURE 3:**
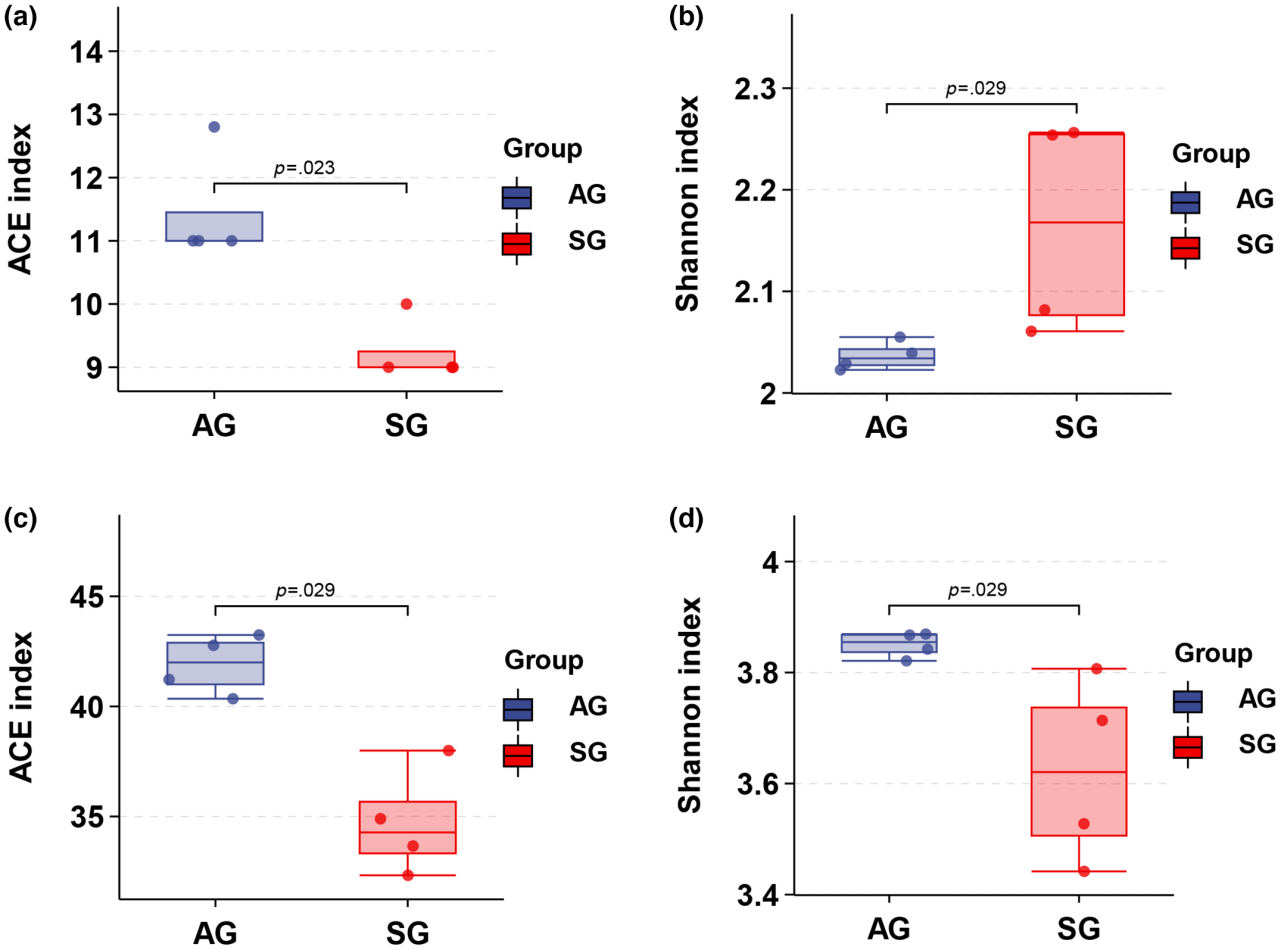
Alpha diversity of gut microbiota from *Teratoscincus roborowskii*. (a) ACE index at Phylum level. (b) Shannon index at Phylum level. (c) ACE index at Family level. (d) Shannon index at Family level. Significant differences between SG (spring) and AG (autumn) groups were tested by Wilcoxon rank‐sum test.

The results of the alpha diversity analysis using the Wilcoxon rank‐sum test at the phylum level showed that the ACE richness estimator in AG (*p* = .023 < .05) was significantly higher than that in SG (Figure 3a). However, the Shannon–Wiener diversity index in SG (*p* = .029 < .05) was significantly higher than that in AG (Figure 3b). At the family level, the ACE (*p* = .029 < .05) and Shannon (*p* = .029 < .05) indices were significantly higher in AG than in SG (Figure 3c,d). In conclusion, the gut microbes of Turpan wonder geckos changed significantly between spring and autumn. During the transition from spring to autumn, microbial richness increased significantly at phyla and family levels, while phyla level diversity decreased and family level diversity increased.

### 3.3 Analysis of beta diversity of gut microbiota

**FIGURE 4:**
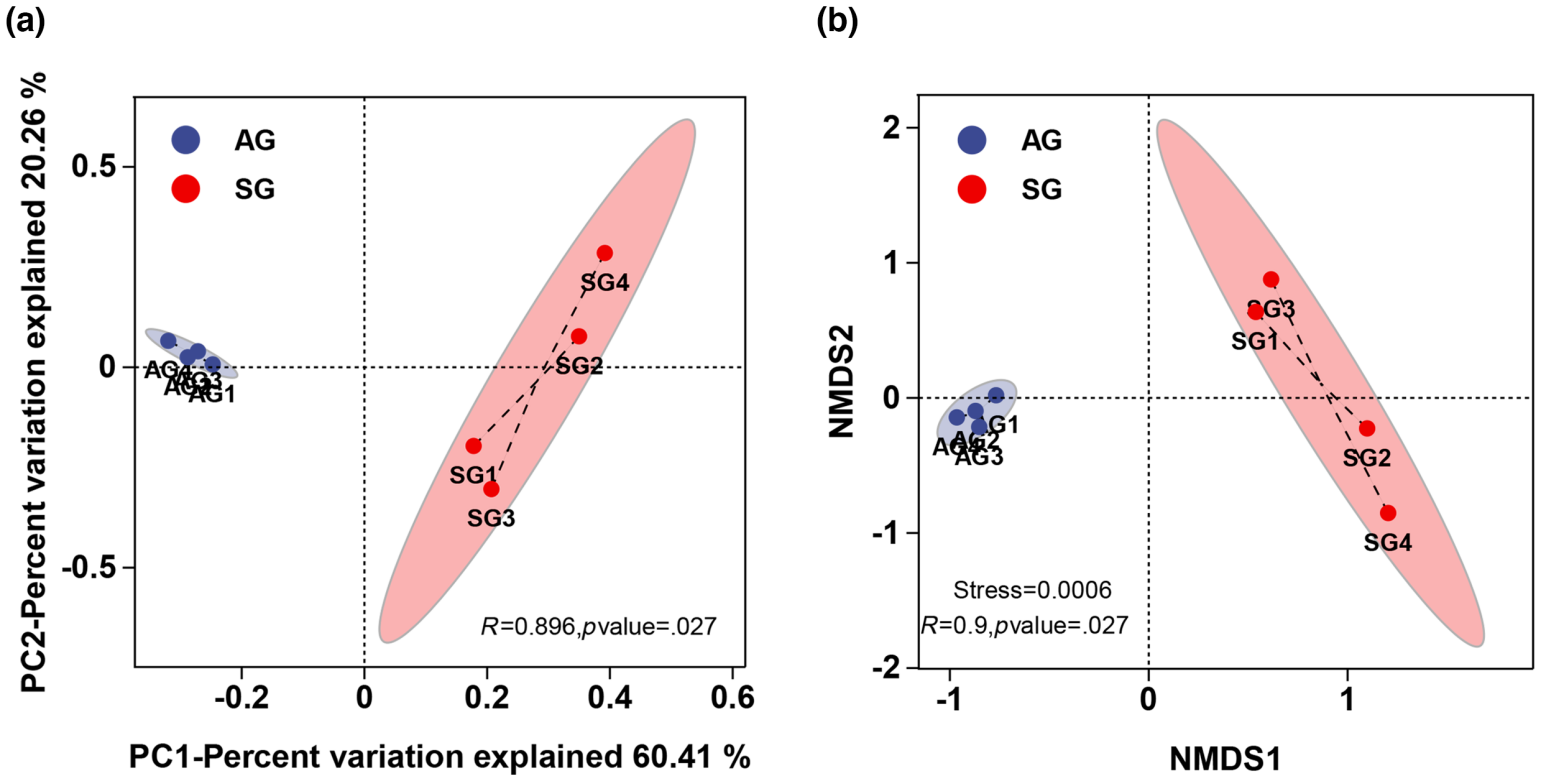
Beta diversity analysis of gut microbiota from *Teratoscincus roborowskii* according to principal coordinates analysis (PCoA) (a) and nonmetric multidimensional scaling (NMDS) (b). (a) Bray‐Curtis distance matrix PCoA of the gut microbiota in the SG (spring) and AG (autumn) groups. The figure shows the discrete distribution of samples along the PC1 and PC2 axes. (b) Nonmetric multidimensional scaling analysis (NMDS) of the Bray‐Curtis distance of the two groups. The *p*‐value was based on ANOSIM.

PCoA was conducted to observe differences in the gut microbial community structure. The results showed that the contribution rates of the first and second principal components were 60.41% and 20.26%, respectively. The gut microbiota composition differed significantly between the spring and autumn groups of *T. roborowskii* according to the Bray–Curtis dissimilarity matrix (PERMANOVA: *R* ^2^ = 0.581; *p* = .001; Anosim: *R* = 0.896; *p* = .0027). According to the similarity analysis results, it is not difficult to see that the distance between the two groups is large and the distance within the group is small. In other words, the samples from the two treatment groups were well clustered, and the separation between the different groups was clear (Figure 4a).

The results of NMDS and similarity analyses indicated a significant difference in the gut microbiota between the spring and autumn groups (stress = 0.0006; Anosim: *R* = 0.9, *p* = .027). The distribution within the group was more discrete in the spring group and more uniform in the autumn group, suggesting that food and environmental influences after hibernation promoted the clustering and stability of the gut microbes in Turpan wonder geckos (Figure 4b).

### 3.4 Analysis of gut microbiota species composition and differences

**FIGURE 5:**
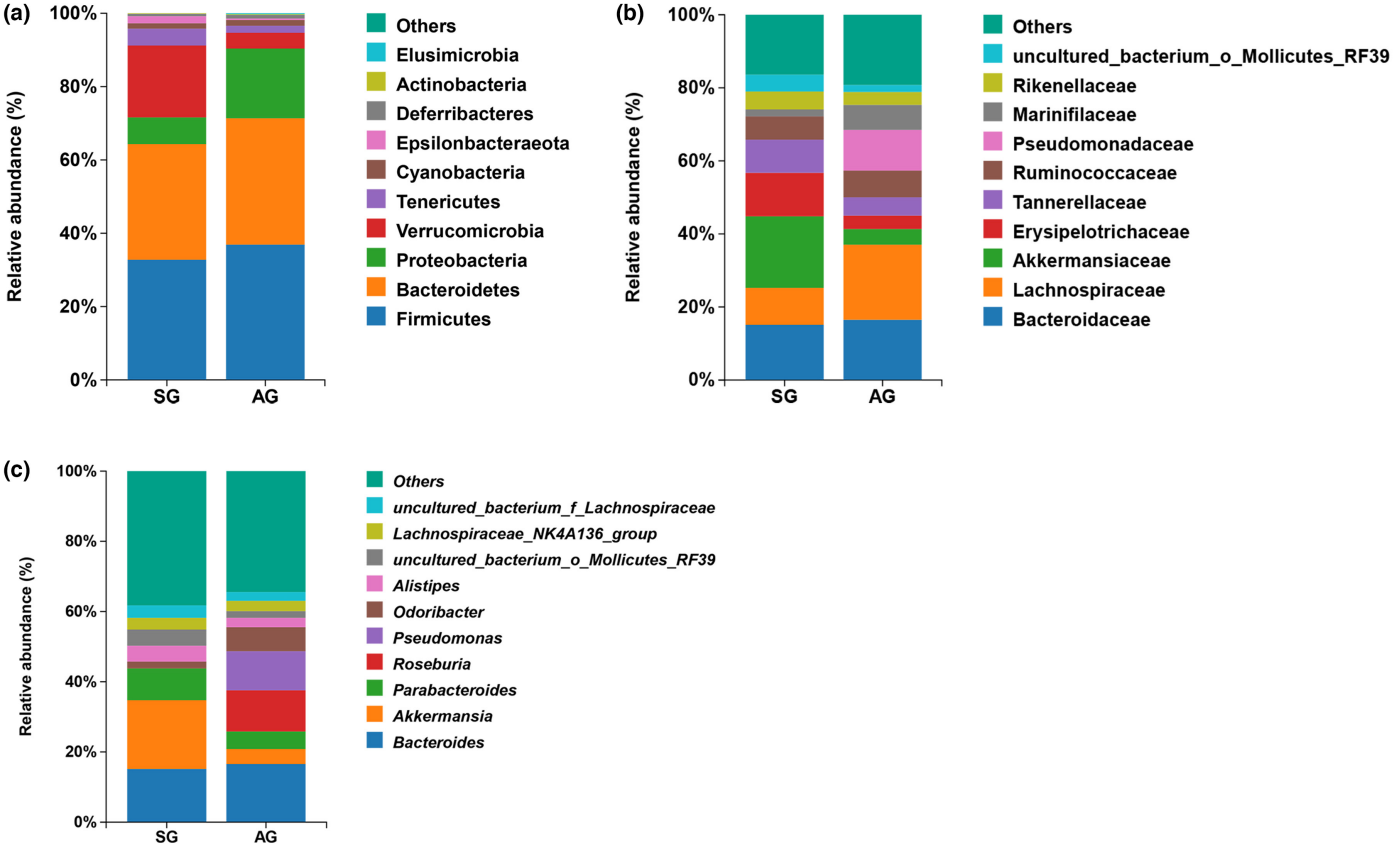
The relative abundance of gut microbiota at the phylum (a), family (b), and genus (c) levels between SG (spring) and AG (autumn) groups in *Teratoscincus roborowskii*. The histogram shows the top 10 bacterial phyla, families, and genera with relative abundance. The vertical coordinates of the figure indicate the relative abundance of bacterial species.

The composition of the gut microbiota in SG and AG of *T. roborowskii* is shown in Figure 5. At the phylum level, the main annotated groups were Firmicutes (32.79%; 36.92%), Bacteroidetes (31.53%; 34.45%), Proteobacteria (7.28%; 18.99%), Verrucomicrobia (19.58% and 4.30%), and Tenericutes (4.62% and 1.93%, respectively; Figure 5a).

At the family level, the main annotated groups were Bacteroidaceae (15.15%; 16.54%), Lachnospiraceae (10.12%; 20.54%), Akkermansiaceae (19.58%; 4.30%), Erysipelotrichaceae (11.90%; 3.67%), Tannerellaceae (9.06%; 4.99%), Lactococcaceae (6.38%; 7.31%), Pseudomonas (0.00%; 11.15%), and others were highly abundant in fecal samples (Figure 5b).

*Bacteroides* (15.15%; 16.54%), *Akkermansia* (19.58%; 4.30%), *Parabacteroides* (9.06%; 4.99%), *Roseburia* (0.06%; 11.72%), *Pseudomonas* (0.00%; 11.15%), *Odoribacter* (1.89%; 6.83%), and *Alistipes* (4.52%; 2.68%), among others, were the dominant bacterial genera of *T. roborowskii* (Figure 5c).

Based on the above analysis of community structure and species composition, the gut microbes of Turpan wonder geckos changed to a certain extent in different seasons. We used LEfSe analysis based on the effect pattern of LDA (LDA > 4, *p* < .05) to screen for microorganisms with significant differences in relative abundance between the two groups. At the phylum level, the relative abundance of Tenericutes (LDA = 4.08, *p* = .02) was significantly higher in SG than in AG; however, the abundance of Proteobacteria was higher in AG (LDA = 4.74, *p* = .02).

At the family level, Erysipelotrichacea (LDA = 4.64, *p* = .021), Tannerellaceae (LDA = 4.31, *p* = .02), and the uncultured bacterium *Mollicutes* RF39 (LDA = 4.06, *p* = .02) were present at significantly higher levels in SG than in AG. In AG, Enterobacteriaceae (LDA = 4.22, *p* = .018), Marinifilaceae (LDA = 4.41, *p* = .02), Lachnospiraceae (LDA = 4.67, *p* = .021), and Pseudomonadaceae (LDA = 4.68, *p* = .014) were significantly more prevalent than in SG.

**FIGURE 6:**
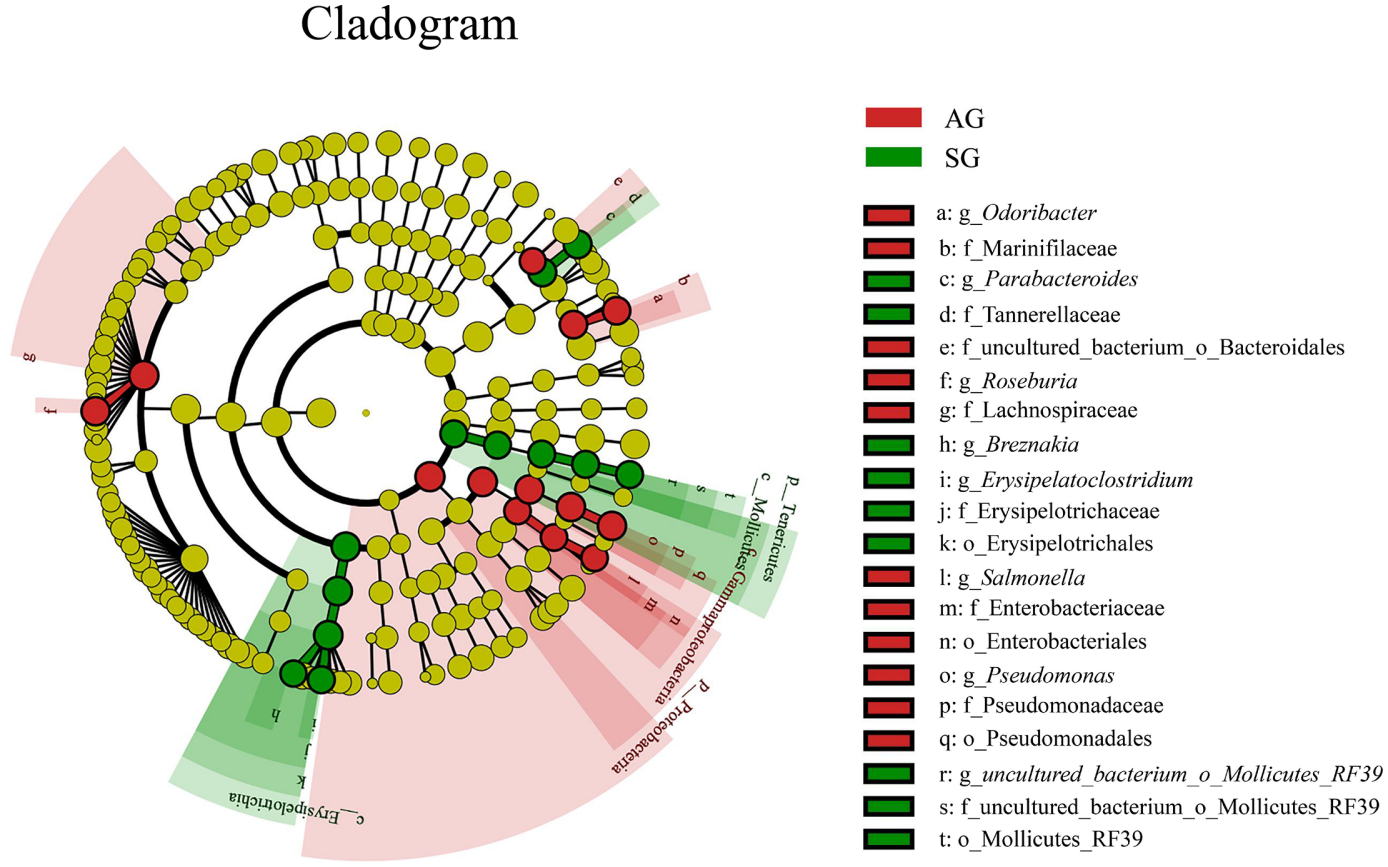
Linear discriminant analysis effect size (LEfSe) analysis of gut microbiota composition between seasons for *Teratoscincus roborowskii* (LDA > 4, *p* < .05). Red boxes and green boxes represent enrichment in the AG (autumn) and SG (spring) groups. Circles radiating from the inside to the outside represent taxonomic levels from phylum to species. Each circle's diameter is proportional to the taxon's abundance. The letters p, o, f, g, and s represent phylum, order, family, genus, and species, respectively.

At the genus level, *Erysipelatoclostridium* (LDA = 4.64, *p* = .02), *Parabacteroides* (LDA = 4.28, *p* = .021), *Breznakia* (LDA = 4.10, *p* = .021), and uncultured bacterium *Mollicutes* RF39 (LDA = 4.10, *p* = .021) were significantly higher in SG, while *Odoribacter* (LDA = 4.38, *p* = .021), *Rothella* (LDA = 4.73, *p* = .014), *Pseudomonas* (LDA = 4.71, *p* = .014), and *Roseburia* (LDA = 4.75, *p* = .021) were significantly higher in AG (LDA > 4, *p* < .05; Figure 6).

### 3.5 Predictive analysis of gut microbial function

The functional pathway abundance of KEGG, corresponding to the 16S rRNA sequencing data, was predicted using PICRUSt2. The results showed that the KEGG metabolic pathways at the first level comprised the following functional categories: metabolism (78.6%), genetic information processing (8.0%), environmental information processing (6.5%), cellular processes (3.0%), human diseases (2.5%), and organic systems (1.4%).

**FIGURE 7:**
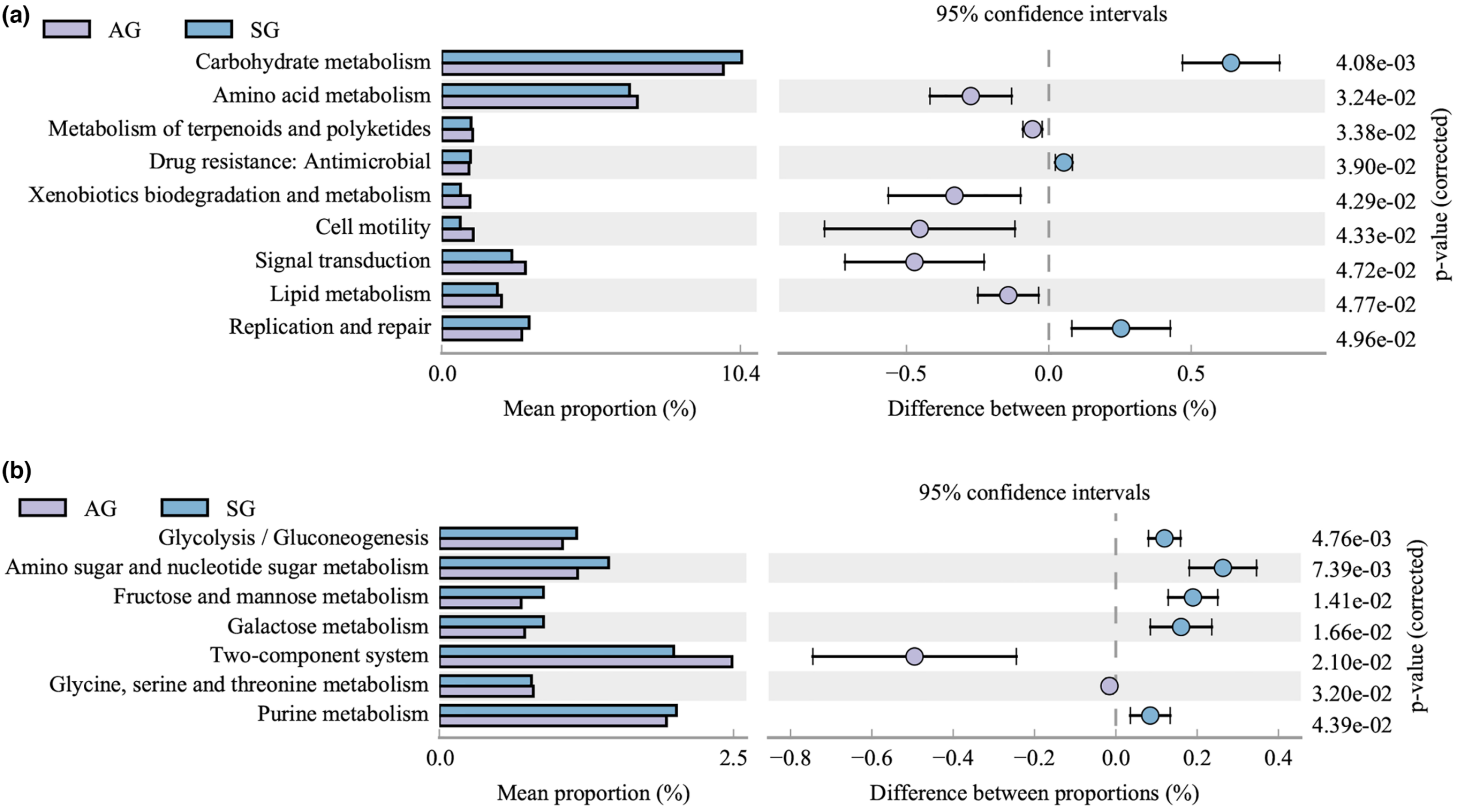
Differential analysis of metabolic functional pathways predicted by KEGG in the AG and SG groups. Significantly‐changed pathways between SG (spring) and AG (autumn) groups at the secondary (a) and tertiary (b) levels (metabolism pathways). The vertical axis represents different pathways, and the horizontal axis represents the proportions of corresponding pathways (*p* < .05).

In the differential analysis of the KEGG metabolic pathways at the second level, the carbohydrate metabolism, replication and repair, and drug resistance pathways were significantly enhanced in SG (*p* < .05). Amino acid metabolism, terpenoid and polyketide metabolism, xenobiotic biodegradation and metabolism, cell motility, signal transduction, and lipid metabolism were significantly enhanced in AG (*p* < .05; Figure 7a).

In the enrichment process of the third‐level metabolic pathways, glycolysis/gluconeogenesis, amino sugar and nucleotide sugar metabolism, and metabolic pathways, such as fructose, mannose, galactose, and purine metabolism, were significantly higher in SG (*p* < .05). The two‐component system and glycine, serine, and threonine metabolism were significantly enhanced in AG (*p* < .05; Figure 7b).

### 3.6 Fecal metabolic profiling of *T. roborowskii*

**FIGURE 8:**
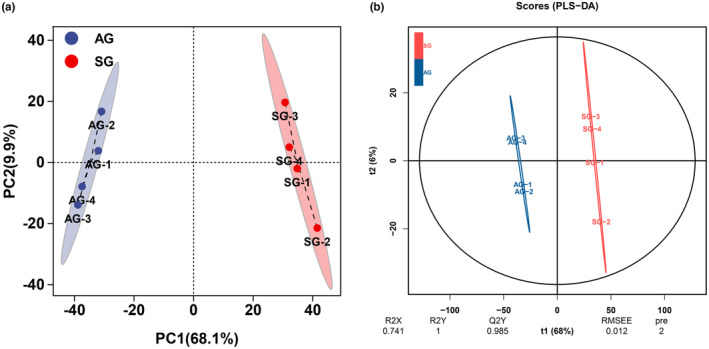
Principal component analysis (PCA) (a) and partial least‐squares discrimination analysis (b) of fecal metabolites in *Teratoscincus roborowskii*. AG1‐4 are fecal metabolites of *T. roborowskii* from autumn group. SG1‐4 are fecal metabolites of *T. roborowskii* from spring group.

The fecal metabolic profiles of *T. roborowskii* were acquired by LC–MS, and metabolites were detected with good intra‐group reproducibility. Fecal metabolites were significantly separated in the first principal component (PC1: 68.1%; Figure 8a). In the OPLS‐DA model, the metabolic curves were clearly different between the two sample groups (Q2 = 0.99 > 0.9, indicating an excellent model), and the results showed obvious differences in fecal metabolites between SG and AG (Figure 8b).

**FIGURE 9:**
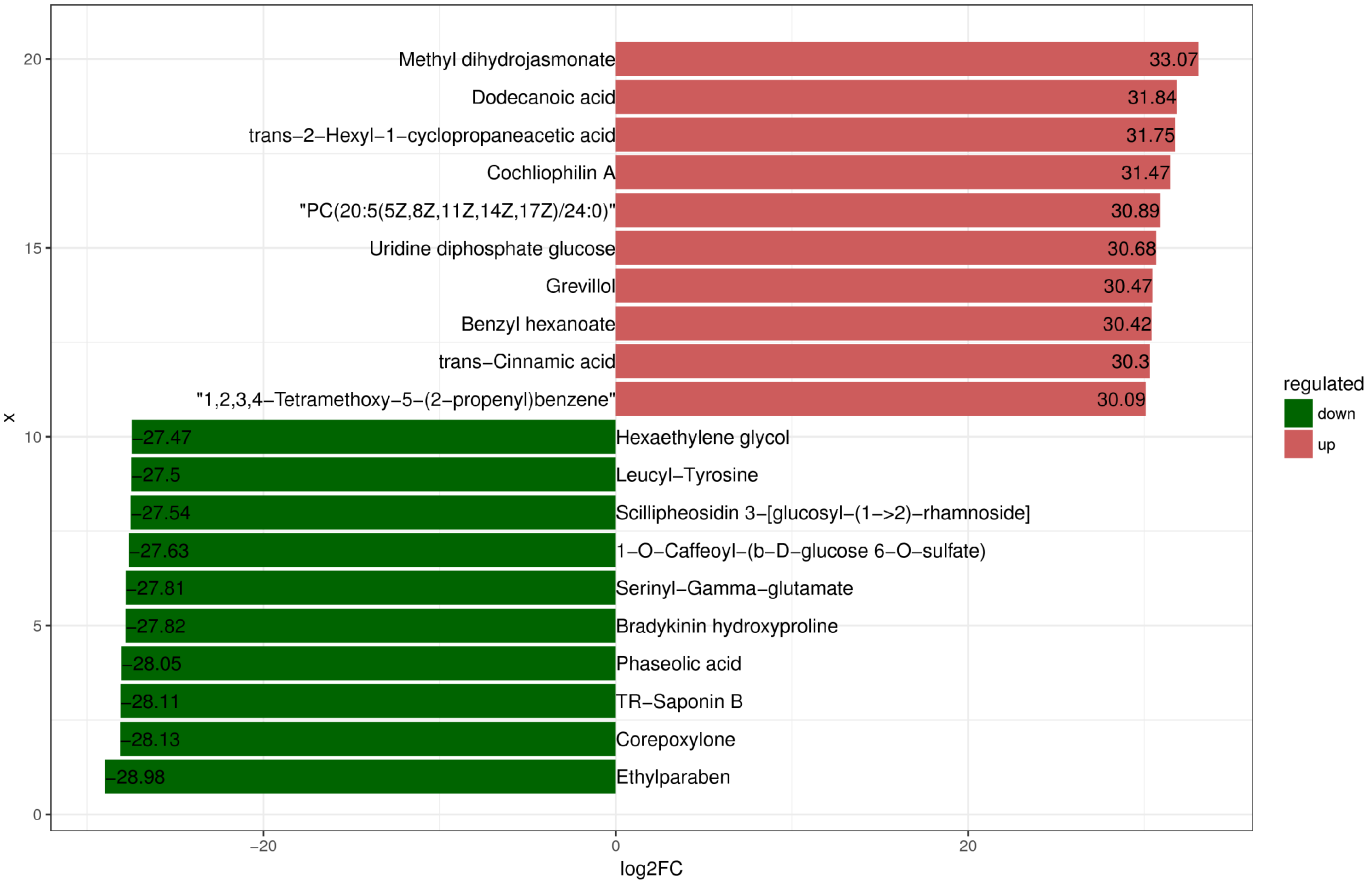
Fold change analysis of fecal metabolite differences based on log2FC size ordering. The figure shows the top 10 metabolites of up and down logFC, and the labels of each column indicate the name of the metabolite. Red bars indicate higher levels in the AG (autumn) group and green bars indicate higher levels in the SG (spring) group.

The absolute values of log2FC (Fold Change, FC) were sorted to get the top 10 metabolites in each group. The levels of hexaethylene glycol, leucyl‐tyrosine, scillipheosidin 3‐[glucosy‐(1‐>2)‐rhamnoside], 1‐O‐caffeoyl‐(b‐D‐glucose 6‐O‐sulfate), serinyl‐gamma‐glutamate, bradykinin hydroxyproline, phaseolic acid, TR‐saponin B, corepoxylone, and ethylparaben were higher in SG. Methyl dihydrojasmonate, dodecanoic acid, trans‐2‐hexyl‐1‐cyclopropaneacetic acid, cochliophilin A, “PC(20:5(5Z,8Z,11Z,14Z,17Z)/24:0),” uridine diphosphate glucose, grevillol, benzyl hexanoate, trans‐cinnamic acid, etc. were higher in AG (Figure 9).

**FIGURE 10:**
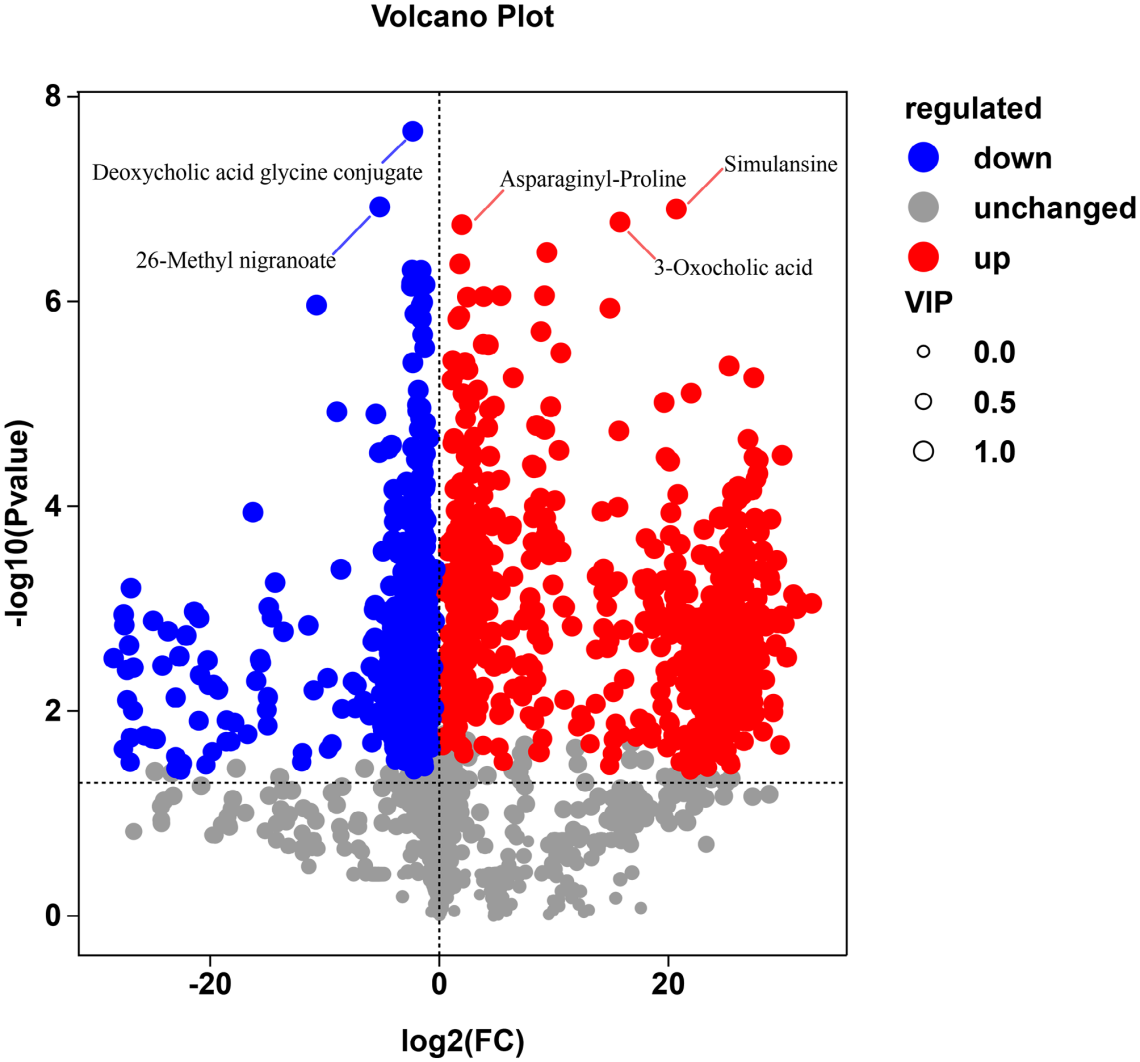
Volcano plot for statistical analysis of fecal differential metabolites. Each point in the volcano plot represents a metabolite, the abscissa represents the multiple changes of the group compared with each substance, the ordinate represents the *p*‐value of the *t*‐test, and the size of the scatter represents the VIP value of the OPLS‐DA model. The blue dots in the figure represent the downregulated differential expression metabolites, which are significantly higher in the spring, and the red dots represent the upregulated differential expression metabolites, which are significantly higher in the autumn. The gray dots represent the detected but not significantly different metabolites. In addition, the top five qualitative metabolites are selected and marked in the figure after sorting by *p* value. The red dots represent metabolites that are significantly higher in the autumn, and the blue dots represent metabolites that are significantly higher in the spring.

The differential metabolites were classified as carboxylic acids and derivatives, fatty acids, organo‐oxygen compounds, steroids and steroid derivatives, glycerol glycerophospholipids, prenol lipids, and glycerolipids. Differential metabolites were screened based on the criteria of *p* = .05, VIP = 1, and FC = 1. Finally, 1264 differential metabolites were detected in the two sample groups, of which 668 were upregulated (higher in AG) and 596 were downregulated (higher in SG). The relative contents of deoxycholic acid glycine conjugate and 26‐methyl nigranoate were higher in SG. Simulansine, 3‐oxocholic acid, and asparaginyl‐proline were the three metabolites with the highest relative content in AG (Figure 10).

**FIGURE 11:**
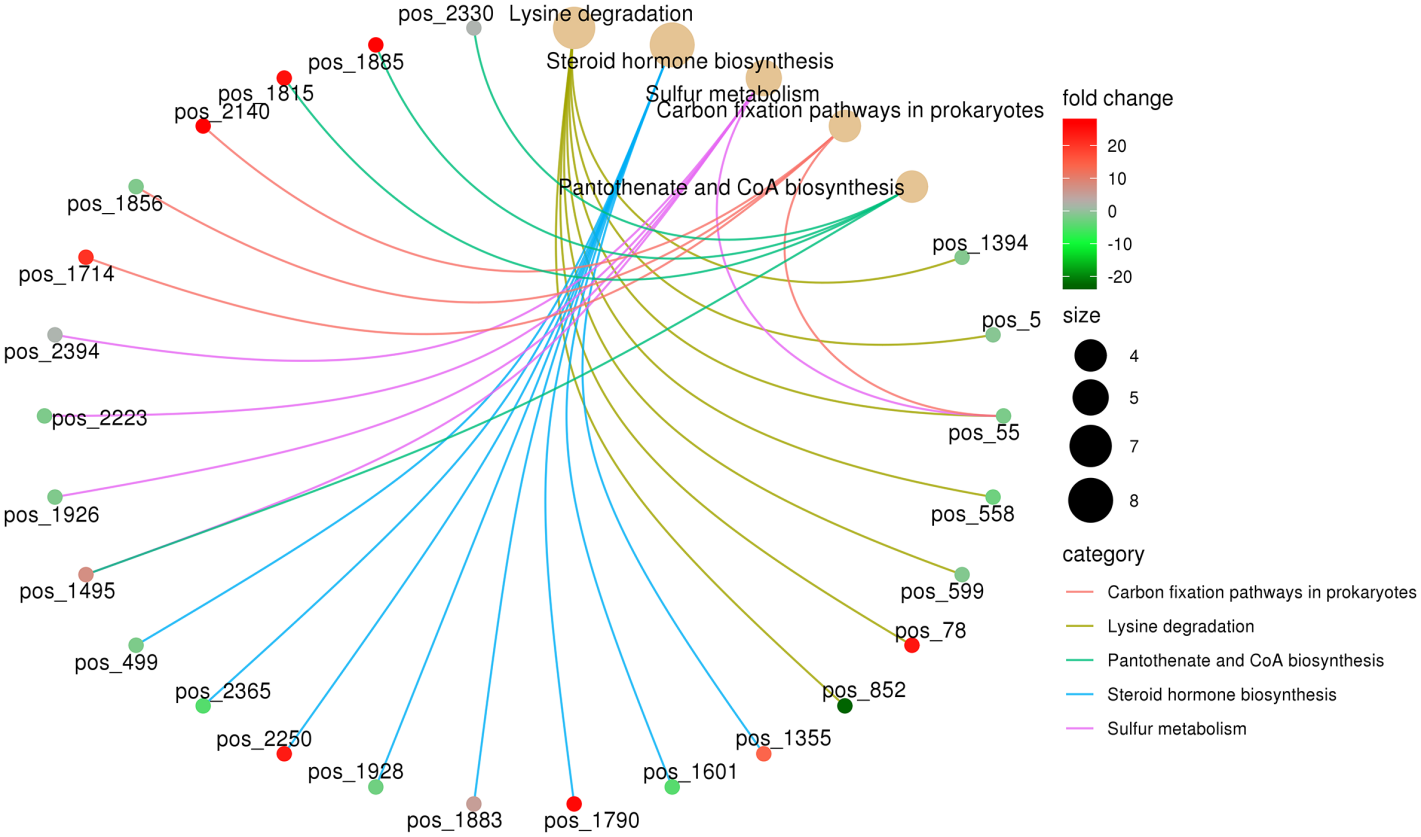
Enrichment of functional pathways corresponding to differential metabolites. The light yellow node in the figure is the pathway, and the small node connected with it is the specific metabolite annotated to the pathway. The value of log2 in the difference multiple determines the color depth.

The KEGG functional classification of differential metabolites showed that they mainly participate in organismal systems, human diseases, environmental information processing, and other functional pathways. Among these, metabolic pathways, biosynthesis of secondary metabolites, and microbial metabolism in different environments are associated with a variety of differential metabolites. The KEGG enrichment network map of differential metabolites showed that lysine degradation, pantothenate and CoA biosynthesis, steroid hormone biosynthesis, carbon fixation pathways in prokaryotes, and other differential metabolites between the different seasons had close positive or negative relationships (Figure 11).

### 3.7 Fecal metabolic profiling and its correlation with the gut microbiota

**FIGURE 12:**
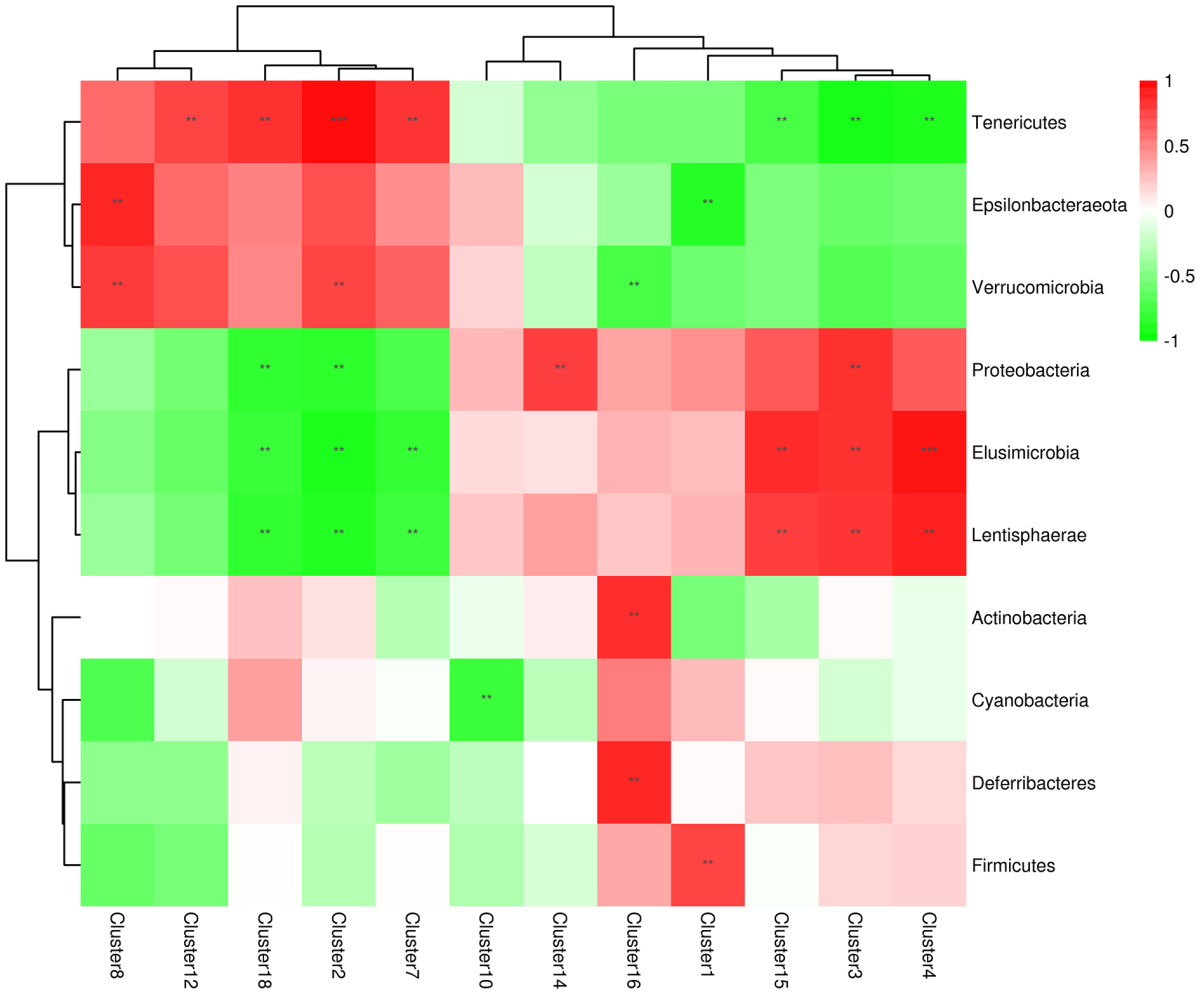
Heat map of the correlation between metabolite clusters and gut microbiota in *Teratoscincus roborowskii*. Different colors represent the size of the Pearson correlation coefficient. Red indicates a positive correlation, whereas green indicates a negative correlation. Asterisks indicate significant correlations between metabolite clusters and microorganisms (*p* < .05); *, **, and *** indicate *p* < .05, *p* < .01, and *p* < .001, respectively.

Metabolite clusters were obtained using hierarchical cluster analysis (HCA). Pearson's correlation analysis of significantly different metabolites and OTUs (microbes) was performed to determine the relationships between the metabolites and microbes (Figure 12). The results showed that Proteobacteria, Verrucomicrobia, Lentisphaerae, Elusimicrobia, and Tenericutes were most closely related to the metabolites, and the dominant microorganisms showed different positive and negative correlations with different metabolite clusters. Among them, Proteobacteria, Lentisphaerae, and Elusimicrobia were relatively abundant in AG, whereas Verrucomicrobia and Tenericutes were relatively abundant in SG. This confirms the results of the LEfSe analysis, and the phyla with significant differences between seasons were significantly correlated with some specific metabolites, suggesting that such synergistic changes are favorable factors for Turpan wonder geckos to cope with seasonal and dietary changes.

## 4 DISCUSSION

*Teratoscincus roborowskii* has evolved a range of adaptive behaviors to escape hot and arid desert environments. For example, the strict nocturnal activity rhythm of *T. roborowskii* (Song et al., 2009) and their special burrow construction not only avoid high temperatures during the day, but also create a relatively constant living temperature. These behaviors are effective mechanisms for adapting to harsh and hot desert environments (Song et al., 2017). Seasonal dietary shifts are also a way of coping with drought environments and enriching the food composition of *T. roborowskii*, which may be the main reason for the change of gut microbiota diversity in *T. roborowskii* between spring and autumn seasons. In the spring group, Verrucomicrobia accounted for 19.85%, belonging to the dominant bacteria, which could prevent the colonization of other new groups through high density, similar to the “Founder takes all” effect (Waters et al., 2013), resulting in the Shannon–Wiener diversity index for phylum of the spring group was significantly higher than that of the autumn group. Proteobacteria in the autumn group may occupy ecological niche rapidly during the seasonal transition, thus breaking the “founder takes all” effect and proliferate. The types of Proteobacteria in the autumn group are more diverse, resulting in the Shannon–Wiener diversity index for family of the autumn group is significantly higher than that of the spring group.

The composition of the gut microbiota in lizards was influenced by dietary shifts (Baldo et al., 2023; Montoya‐Ciriaco et al., 2020). Kohl et al. (2016) fed *Liolaemus ruibali* a diet ratio of 50% insects +50% plants or 10% insects +90% plants and found that lizards fed a rich plant‐based diet showed a higher level of gut microbial diversity. This is consistent with our results, that is, the intake of caper fruit significantly increased the diversity of gut microbes, and this change may be beneficial to the achievement of gut function and the stabilization of microbiota in *T. roborowskii*.

At the phylum level, Firmicutes, Bacteroidetes, Proteobacteria, and Verrucobacterium in the gut microbes of *T. roborowskii* in spring and autumn accounted for more than 91% of all groups and were the dominant phyla. Studies have shown that Firmicutes, Bacteroidetes, and Proteobacteria are important members of the gut microbiota in most vertebrates (Hale et al., 2019; Kohl et al., 2013; Wang et al., 2018; Zhao et al., 2018). As a nocturnal desert gecko, the leopard gecko (*Euplepharis macularus*) possesses a gut microbial composition highly similar to that of *T. roborowskii*, and remains stable after 28 days of fasting, suggesting that animals living in extreme desert environments have a high degree of control over gut microbes (Kohl et al., 2014). Other lizard species, such as the toad‐headed lizard (*Phrynocephalus vlangalii*; Zhang et al., 2018), *S. crocodilurus* (Jiang et al., 2017), *T. septentrinalis* (Zhou et al., 2020), and *S. occidentalis* (Moeller et al., 2020), are also dominated by Firmicutes and Bacteroidetes, which indicates that different lizard taxa have similar gut microbial compositions at higher taxonomic levels.

However, the gut microbial composition of *T. roborowskii* differs from, and converges somewhat with, that of other lizard taxa at the family and genus levels. *S. crocodilurus* lives in warm and moist habitats and mainly feeds on earthworms and loaches. The main bacterial families found were Pasteurellaceae, Deinococcaceae, and Comamonadaceae (Jiang et al., 2017). There were significant differences compared with *T. roborowskii*. A study of *T. septentrionali*s from eastern China showed that this insect‐eating lizard has a gut microbiota composition similar to that of *T. roborowskii* at the family level; however, its abundance differs greatly among families (Zhou et al., 2020). *P. vlangalii*, living at high altitudes, appears to have a gut microbial composition more similar to that of *T. roborowskii* (Zhang et al., 2018), which may be due to similar food sources and arid environments. It can be seen that some factors such as diet (Kohl et al., 2016) and environmental factors (Moeller et al., 2020) can significantly affect the composition of lizards' gut microbes.

Firmicutes and Bacteroidetes are two of the most important gut microbiota types of Turpan wonder geckos both in spring and autumn, which is consistent with other studies on the microbiome of lizards (Littleford‐Colquhoun et al., 2019; Montoya‐Ciriaco et al., 2020; Zhang et al., 2018). Bacteroidetes can ferment amino acids and carbohydrates and are involved in bile acid, polysaccharide, and steroid metabolism (Rios‐Covian et al., 2017), which function together to promote fat accumulation (Turnbaugh et al., 2006). The relative stability of the dominant phyla may be favorable for maintaining a high nutrient absorption capacity during the active season. On the one hand, this meets the direct cost of summer breeding input (Speakman, 2008), and on the other hand, it ensures energy accumulation.

Akkermansiaceae are widely present in the gut of hibernating animals (Tang et al., 2019), and are considered probiotics with functions such as promoting intestinal mucosal barrier repair and regulating intestinal flora metabolism (Belzer & De Vos, 2012). The relative abundance of Akkermansia muciniphila was significantly higher in SG than in AG. We speculate that in the period in which it was collected, *T. roborowskii* was still in the transitional period of enterotype transition; therefore, these individuals still had a higher abundance of Verrucobacterium. Akkermansiaceae can produce short‐chain fatty acids such as acetate and propionate by degrading intestinal mucin (Feng et al., 2018). The production of fatty acids can maintain the intestinal immune state and create the anaerobic environment required for the growth of strictly anaerobic symbiotic microorganisms, thereby establishing a mutually beneficial relationship with the host (Shealy et al., 2021). Most Enterobacteriaceae and Pseudomonas are facultative anaerobes, and destruction of the anaerobic environment leads to their rapid proliferation (Rivera‐Chávez et al., 2016). Therefore, the high abundance of Verrucomicrobia in spring limited Proteobacteria; however, the abundance of Verrucomicrobia decreased in autumn, and certain aerobic Proteobacteria dominated.

Relevant studies have shown that Proteobacteria are related to a variety of metabolic pathways and can secrete a large number of enzymatic substances related to polysaccharide and protein metabolism, which can effectively decompose polysaccharides and vitamins (Abdul Rahman et al., 2016; Colston & Jackson, 2016). In our study, the richness of Proteobacteria in AG was significantly higher than that in SG (*p* < .05), this may be largely due to the special frugivorous behavior of *T. roborowskii* in the long‐term evolutionary. Furthermore, it was speculated that the richness of Proteobacteria was in an effort to the digestion of caper fruits and insects in autumn, which improve the utilization rate of food resources. Related studies have shown that some Enterobacteriaceae species possess sucrose‐specific phosphotransferase systems and sugar transporters with different functions and structures (Le Bouguénec & Schouler, 2011). Carbohydrate metabolism is also widely recognized as the nutritional basis for γ‐proteobacteria to colonize the gut and maintain strains (Chang et al., 2004). The relative abundances of Enterobacteriaceae and *Pseudomonas* in AG were significantly higher than those in SG, which may also be the result of adaptation to fruit foods with higher sugar content.

*Lachnospira* and Ruminococcaceae were the most abundant Firmicutes in the gut of *T. roborowskii*. Most studies have confirmed that both contain abundant genes related to polysaccharide degradation and that their ability to utilize dietary polysaccharides is effective (Vacca et al., 2020). The high abundance of ABC transporters is the basis for the utilization of complex plant materials and the transport of various degradation products (Biddle et al., 2013), which utilizes lactic acid and acetate to produce short‐chain fatty acids, such as butyrate, through the butyryl‐CoA: acetate CoA‐transferase pathway (Flint et al., 2015). The significant increase in Lachnospiriaceae (*Roseburia*) in autumn may be an adaptation to the abundant heteropolysaccharides in caper fruits (Bai et al., 2007), and may improve the efficiency of nutrient metabolism.

Analysis based on PCA and PLS‐DA showed significant differences in fecal metabolites among different seasons, which may be caused by differences in seasonal diets (Li et al., 2020). Plant‐derived chemicals such as lauric acid and cinnamic acid were significantly enriched in AG because *T. roborowskii* had a limited ability to digest caper seeds (Yang et al., 2021), and some plant‐derived components were retained. Deoxycholic acid glycine conjugate and 3‐oxocholic acid are secondary bile acids. Bile acids participate in cholesterol metabolism through the enterohepatic circulation. This plays a crucial role in the digestion and absorption of components, regulates carbohydrate metabolism, and effectively regulates metabolic homeostasis (Bao et al., 2016).

CoA is involved in various biochemical reactions including the tricarboxylic acid cycle, fatty acid synthesis and oxidation, and amino acid metabolism (Choudhary et al., 2014; Ma et al., 2020). Our results showed that the biosynthetic pathways of pantothenate and CoA were significantly associated with Pantothenate and D‐4′‐phosphopantothenate, which were the precursors of pantothenate and CoA. High CoA biosynthesis in autumn suggests that *T. roborowskii* may have improved their metabolic capacity. Pipecolic acid pathway was enriched in the SG samples. This was correlated with higher levels of the L‐piperic acid, 5‐aminopentanoicacid, and D‐1‐piperideine‐2‐carboxylic acid, which indicates that microbiota involved in this pathway may lead to greater metabolism of them, a dominant component of caper fruit. Furthermore, it was shown that L‐lysine improves the absorption and utilization of food proteins (Hallen et al., 2013). This may explain the ability of Turpan wonder geckos to cope with a protein‐rich, insect‐based diet in spring.

Correlation analysis between metabolite clusters and gut microbes revealed that Verrucomicrobia and Tenericutes were more abundant in spring, whereas Proteobacteria and Elusimicrobia were more abundant in autumn. Verrucomicrobia, Tenericutes, and Epsilonbacteraeota have a positive correlation with a variety of metabolic clusters, which means that they have a certain synergistic ability when performing metabolic functions. The same synergistic relationship also exists among Proteobacteria, Elusimicrobia, and Lentisphaerae, but it is completely different from the related metabolites such as Verrucomicrobia, suggesting that there is a certain antagonistic relationship between them. This change was correlated with a variety of metabolic clusters. Although we are not sure whether the metabolites are directly derived from the microbiota, this change in the microbiota and metabolites is undoubtedly the result of adaptation to seasonal and dietary changes. Further studies are required to determine how these altered microbes crosstalk with each other and influence the hosts' metabolic function.

## 5 CONCLUSION

In summary, seasonal changes were observed in the gut microbes of *T. roborowskii*, and the abundance of phylum level in autumn was significantly higher than spring, but the diversity was lower. At the family level, the abundance and diversity of the gut microbiota were both higher in autumn. The proportions of Firmicutes and Bacteroidetes were stable. Proteobacteria and the significant growth of Lachnospiriaceae in autumn may be an adaptive feature for coping with seasonal dietary shifts. Secondary bile acids, such as deoxycholate‐glycine conjugates, showed higher levels in different seasons, suggesting their important roles in metabolism. PICRUSt2 functional predictions showed that metabolism‐related pathways varied in different seasons and that the core microbiota in different seasons appeared to perform functions associated with specific metabolic pathways.

Additionally, the approach of mixing fecal samples was adopted in this study, however, the mixed samples and the unequal numbers of fecal samples were mixed in each season may be a potential factor that caused more microbiota diversity in the AG group than that of the SG group, as the higher the number of individuals included, the higher the number of transient or individual specific taxa likely to be captured is. In contrast, the mixed samples might conceal the gut microbiota of individual characteristics, so for further study we should increase the number of samples and analyze the changes in gut microbiota and metabolism at the individual level. In the future, captive breeding and artificial manipulation of the food ratio will be effective ways to further understand seasonal dietary shifts and the effects of frugivory strategies on the gut microbiota and metabolic functions of lizards such as *T. roborowskii*.

## AUTHOR CONTRIBUTIONS

**Lei Shi:** Data curation (equal); investigation (equal); methodology (equal); software (equal); supervision (equal); writing – original draft (equal); writing – review and editing (equal). **Wei‐Zhen Gao:** Data curation (equal); investigation (equal); methodology (equal); software (equal); supervision (equal); writing – original draft (equal). **Yi Yang:** Data curation (equal); methodology (equal); software (equal); writing – original draft (equal); writing – review and editing (equal).

## FUNDING INFORMATION

We gratefully acknowledge funding from the National Natural Science Foundation of China (32260527, 32260118, and 31260511) and the Innovative Team Foundation of Biology of Xinjiang Agricultural University, China (ITB202101).

## CONFLICT OF INTEREST STATEMENT

The authors declare that they have no competing interests.

## Supporting information


Appendix S1.

## Data Availability

The data supporting the findings of this study are publicly available from the National Center for Biotechnology Information (NCBI) Sequence Read Archive (SRA) at https://www.ncbi.nlm.nih.gov/bioproject/PRJNA887219, reference number [PRJNA887219].
