# Supplementary material for: Seasonal dietary shifts alter the gut microbiota of a frugivorous lizard Teratoscincus roborowskii (Squamata, Sphaerodactylidae)
Source: Ecol Evol. 2023 Aug 2;13(8):e10363. doi: 10.1002/ece3.10363 (PMC10396791; doi:10.1002/ece3.10363)
Supplement: Supplementary file 1 — Appendix S1. [file ECE3-13-e10363-s001.docx]

Appendix

## Supplementary Table

Supplementary Table 1 Animal and fecal sample collection information

| Spring Group | Gender | Weight  (g) | SVL  (mm) | Fecal number | Sample | Autumn Group | Gender | Weight  (g) | SVL  (mm) | Fecal number |  |
| --- | --- | --- | --- | --- | --- | --- | --- | --- | --- | --- | --- |
| 1 | Male | 14.50 | 73.36 | 2 | SG1 | 1 | Male | 14.23 | 75.41 | 1 | AG1 |
| 2 | Female | 24.71 | 94.72 | 0 |  | 2 | Female | 13.64 | 75.62 | 1 | AG1 |
| 3 | Female | 15.73 | 77.33 | 3 | SG1 | 3 | Female | 20.00 | 88.09 | 1 | AG1 |
| 4 | Female | 20.61 | 87.53 | 3 | SG2 | 4 | Female | 14.95 | 77.70 | 1 | AG1 |
| 5 | Female | 12.60 | 70.36 | 2 | SG2 | 5 | Female | 15.95 | 78.84 | 3 | AG2 |
| 6 | Male | 20.62 | 90.23 | 2 | SG2 | 6 | Female | 15.12 | 78.95 | 2 | AG2 |
| 7 | Female | 16.30 | 86.31 | 1 | SG3 | 7 | Female | 14.53 | 80.95 | 1 | AG2 |
| 8 | Female | 12.44 | 71.23 | 3 | SG3 | 8 | Male | 12.13 | 70.31 | 1 | AG2 |
| 9 | Male | 24.23 | 93.15 | 3 | SG3 | 9 | Female | 18.13 | 82.98 | 1 | AG3 |
| 10 | Male | 20.32 | 89.67 | 2 | SG4 | 10 | Female | 19.25 | 86.08 | 1 | AG3 |
| 11 | Female | 12.81 | 71.08 | 2 | SG4 | 11 | Male | 14.76 | 81.68 | 1 | AG3 |
|  |  |  |  |  |  | 12 | Male | 16.84 | 86.50 | 1 | AG3 |
|  |  |  |  |  |  | 13 | Female | 12.18 | 75.10 | 3 | AG4 |
|  |  |  |  |  |  | 14 | Female | 12.85 | 74.05 | 1 | AG4 |
|  |  |  |  |  |  | 15 | Female | 11.03 | 69.36 | 2 | AG4 |
| Total | |  |  | 23 |  |  |  |  |  | 21 |  |

Note: SVL:snout-vent lengths

Supplementary Table 2 Sample sequencing data information

Note: CCS:Circular Consensus Sequencing
